# Mapping the molecular basis for growth related phenotypes in industrial producer CHO cell lines using differential proteomic analysis

**DOI:** 10.1186/s12896-021-00704-8

**Published:** 2021-07-23

**Authors:** Laura Bryan, Michael Henry, Ronan M. Kelly, Christopher C. Frye, Matthew D. Osborne, Martin Clynes, Paula Meleady

**Affiliations:** 1 National Institute for Cellular Biotechnology, Dublin City University, Glasnevin, Dublin 9, Ireland; 2 Eli Lilly and Company, LTC-North, 1200 Kentucky Avenue, Indianapolis, IN 46225 USA; 3 Eli Lilly, Kinsale Limite, Cork, Ireland

**Keywords:** Chinese hamster ovary (CHO) cells, Label free quantitative proteomics, Cell specific productivity (Qp), Viable cell density (VCD) biopharmaceuticals

## Abstract

**Background:**

The ability to achieve high peak viable cell density earlier in CHO cell culture and maintain an extended cell viability throughout the production process is highly desirable to increase recombinant protein yields, reduce host cell impurities for downstream processing and reduce the cost of goods. In this study we implemented label-free LC-MS/MS proteomic profiling of IgG4 producing CHO cell lines throughout the duration of the cell culture to identify differentially expressed (DE) proteins and intracellular pathways associated with the high peak viable cell density (VCD) and extended culture VCD phenotypes.

**Results:**

We identified key pathways in DNA replication, mitotic cell cycle and evasion of p53 mediated apoptosis in high peak VCD clonally derived cell lines (CDCLs). ER to Golgi vesicle mediated transport was found to be highly expressed in extended culture VCD CDCLs while networks involving endocytosis and oxidative stress response were significantly downregulated.

**Conclusion:**

This investigation highlights key pathways for targeted engineering to generate desirable CHO cell phenotypes for biotherapeutic production.

**Supplementary Information:**

The online version contains supplementary material available at 10.1186/s12896-021-00704-8.

## Background

CHO cells are the most frequently used host cell line for the production of therapeutic proteins [1] due to their ability to produce human like post-translational modifications, their high level of approval among regulatory authorities and their stable transgene expression [2, 3]. Optimising growth, titre and specific productivity of these cells has long been an area of interest among the pharmaceutical industry; however, the vast majority of improvements to date can be attributed to optimised feeding strategies and adaption to serum free medium [4]. Little progress has been made towards understanding the intracellular pathways that contribute to creating industrially desirable phenotypes in CHO cells. With a deeper knowledge of CHO cell biology, cellular engineering strategies can be developed to target pathways and proteins that are associated with phenotypes of interest. The publishing of the CHO genome in 2013 was the first step towards advancing our knowledge of CHO cell biology [5–8]. Most strategies aimed at increasing recombinant protein production focus on achieving high specific productivity while also maintaining a high VCD throughout the cell culture process duration. Parameters such as temperature and medium are understood to play a significant role in the growth and productivity of CHO cells [9–12]. Lowering cell culture temperature has been shown to result in cell cycle arrest at the G0/G1 phase of growth [13, 14] and has been associated with improvements in folding, translation and processing of proteins [15, 16]. Reducing cell culture temperature has also been shown to result in a slowdown of growth and metabolism as indicated by reduced glucose and glutamine consumption [17, 18], reduced lactate and ammonium production [19, 20] and a lower growth rate [21, 22].

Recent advances and applications of cell profiling technologies such as label-free LC MS/MS proteomic analysis has allowed investigators to gain a greater understanding of the key molecular factors and associated pathways in CHO cell biology [23, 24]. Assimilation and interrogation of these data has allowed for targeted identification of the differences at the protein level between desirable and undesirable bioprocess phenotypes in CHO cells [25–28]. Increasing culture VCD and maintaining an extended high VCD in CHO cells are highly desirable phenotypes for retention of cell specific productivity (Qp) and increasing overall recombinant protein titres. CHO cell lines that reach high peak VCDs early in culture are desirable due to their potential to reduce cell culture production duration and allow for increased seeding densities. These improvements in the efficiency of production will ultimately lead to a reduction in the costs of complex biotherapeutics making them more accessible to patients. The effects of increased seeding density on recombinant monoclonal antibody (mAb) production have been previously described [29]. Increased and extended culture VCD can also help reduce negative effects related to the release of intracellular proteases in culture. Proteolytic degradation of secreted polypeptides in culture represents one of the most significant hurdles presented by mammalian host cell lines [30–33]. Attempts to reduce the negative effects of intracellular proteases have included media optimisation, reduced culture temperature, optimised pH and early product harvesting [34–36]. Increased culture VCD can allow for early product harvest in order to reduce the effects of intracellular proteases, however, extended culture VCD could also help to reduce the numbers of proteases that are released into the culture media from non-viable cells. The reduction in protease levels along with other host cell protein (HCP) contaminants has a positive impact on downstream processing steps which involve the removal of process related impurities such as DNA/RNA, lipids and host cell proteins [37]. When recombinant protein titres are high, most of the manufacturing costs become associated with downstream processing [38–40]. Decreased levels of process-related impurities in culture due to increased and extended culture VCD will help reduce the burden on downstream processing steps and in turn lower the costs associated with downstream processing. Maintaining an extended high culture VCD phenotype usually results in the stationary phase of growth being prolonged. The transition of growth from the exponential phase to the stationary phase and ultimately to the death phase together determine the integral viable cell density (IVCD) of the culture. The IVCD of the culture has been shown to be positively correlated with product titre [41].

In this study we identified differentially expressed (DE) proteins and pathways associated with the high peak VCD and extended culture VCD phenotypes and in turn identified potential targets for engineering of these phenotypes in CHO CDCLs. Unfortunately, efforts to create one desirable phenotype in CHO cells can often compromise another. This is evident in temperature shifted CHO cells which experience a lower growth rate in addition to improved protein folding and translation [13, 14]. For this reason, we believe mapping each growth phenotype separately may provide a deeper understanding of the intracellular pathways which influence growth related phenotypes. We therefore selected IgG4 mAb producing CHO CDCLs displaying high peak VCD, low peak VCD, extended culture VCD and normal culture VCD phenotypes from a panel of industrially relevant CHO CDCLs produced in a cell line generation experiment. CDCLs were grown in a 17-day fed batch shake flask study and samples were taken for differential label-free LC-MS/MS proteomic profiling on days 6 and 10 of culture. A unique aspect of this study is the comprehensive characterisation which was applied to all CHO cell CDCLs. This allowed us to identify any additional parameters which could be affecting phenotypes such as cell size, cell volume, transgene copy number or transcript copy number.

## Results

### Phenotypic assessment of IgG4 expressing CHO CDCLs

#### High/low peak VCD CDCLs

**Fig. 1:**
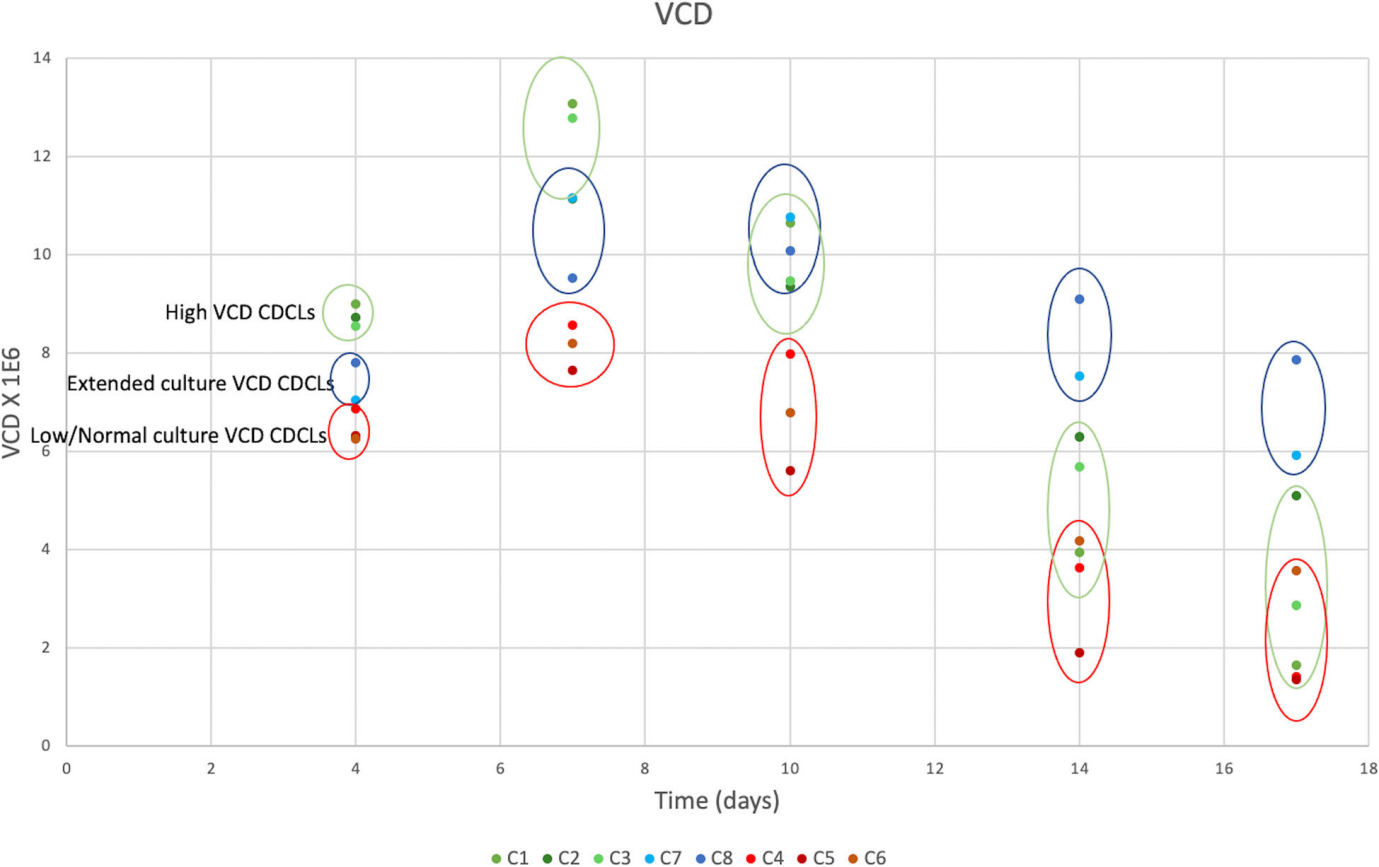
VCD profiling of all CDCLs examined in this cell culture terminal study. Green circles highlight CDCLs deemed as high peak VCD, blue circles highlight CDCLs deemed as having extended culture VCD and red circles highlight the CDCLs which were deemed as low peak VCD/ normal culture VCD

**Fig. 2:**
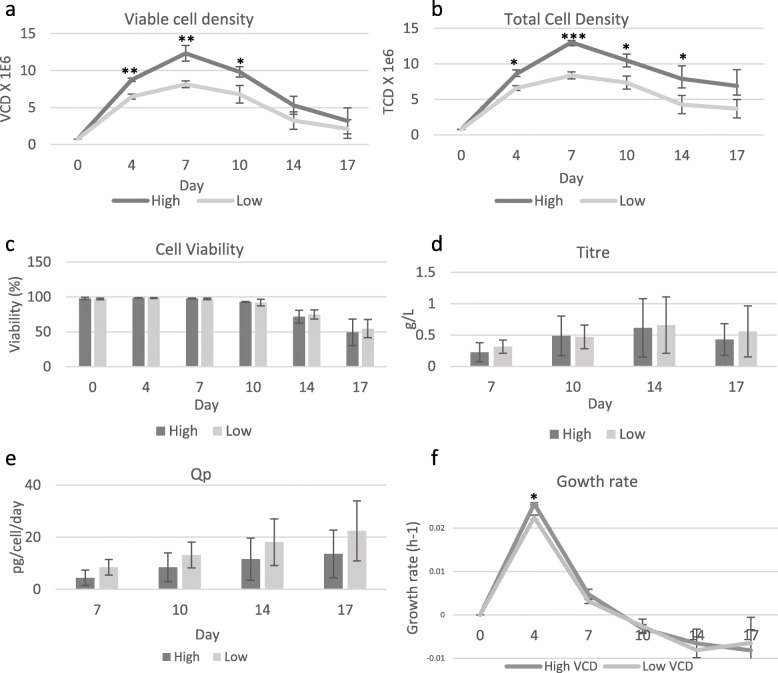
Profiling of high/low peak VCD peak CDCLs throughout the shake flask terminal study. Average (**a**) VCD, (**b**) TCD, (**c**) Cell viability, (**d**) Titre, (**e**) Specific productivity, (**f**) Growth rate (h^−1^) of high peak VCD and low peak VCD CDCLs. Error bars represent the standard deviation of three high peak VCD or three low peak VCD CDCLs, with two biological replicates per CDCL. (* < 0.05, ** < 0.005, *** < 0.001)

High/low peak VCD CDCLs were chosen for differential LC-MS/MS analysis. Growth characteristics were measured on days 0, 4, 7, 10, 14 and 17. CDCLs which reached an observed peak VCD of > 10 × 10^6^ cells/ml were grouped as high peak VCD and < 10 × 10^6^ cells/ml were grouped as low peak VCD. All CDCLs were thoroughly characterised for several phenotypic parameters during the 17-day fed batch shake flask study. High peak VCD CDCLs were found to have peak VCDs of between 11 and 13 × 10^6^ cells/ml. Low peak VCD CDCLs were found to have peak VCDs of between 7 and 9 × 10^6^ cells/ml. High peak VCD CDCLs were also found to have a significantly higher growth rate at day 4. Although peak VCD in these clones was observed at day 7, it should be noted that actual peak VCD may have been reached any time between day 7–9. Figure 1 outlines the growth characteristics of the CDCLs selected for the high versus low peak VCD LC-MS/MS proteomic analysis. Day 6 and 10 time-points were chosen for LC-MS/MS profiling due to all CDCLs maintaining a similarly high viability at these time-points. Day 6 represents the exponential growth phase and day 10 represents the early decline phase of growth for high/low peak VCD CDCLs. High peak VCD CDCLs were found to exhibit a significantly higher VCD and TCD during all stages of growth (Fig. 2). IVCD was found to be higher in high peak VCD CDCLs at days 4, 7, 10 and 14 (Additional file 3). This demonstrates a higher accumulation of viable cells over time in the high peak VCD CDCLs. No significant difference in viability, titre, Qp, cell size, cell volume, gene copy number or transcript copy number was detected between high and low peak VCD CDCLs (Fig. 2 and Additional files 1 and 4). An higher gene copy number was observed in high VCD CDCLs, however, this was not found to be statistically significant due to a high level of variance in gene copy number between high VCD CDCLs. Waste products and metabolites of the cells were measured throughout culture with no significant difference in lactate or ammonia being detected between high and low peak VCD CDCLs (Additional file 3). Glucose levels were found to be higher in low peak VCD CDCLs at day 10 and glutamine levels were found to be significantly higher in low peak VCD CDCLs at all days. Glutamate was measured and used as a indicator of the need for glutamine feeds over the culture duration.

#### Extended/Normal culture VCD

**Fig. 3:**
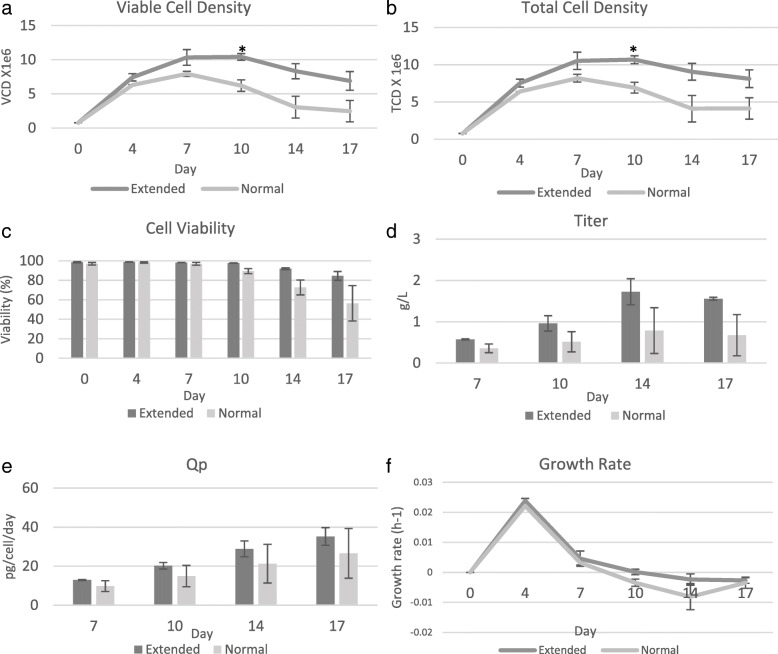
Profiling of extended culture VCD and normal culture VCD CDCLs throughout the shake flask terminal study. Average (**a**) VCD, (**b**) TCD, (**c**) Cell viability, (**d**) Titre, (**e**) Specific productivity, (**f**) Growth rate (h^− 1^) of normal and extended culture VCD CDCLs. Error bars represent the standard deviation of two extended VCD or two normal VCD CDCLs, with two biological replicates per CDCL. (* < 0.05, ** < 0.005, *** < 0.001)

Extended culture VCD CDCLs were chosen based on cells reaching a high VCD and maintaining a high VCD for longer throughout the cell culture process. CDCLs were deemed as having an extended culture VCD if the VCD at day 10 was greater or not significantly lower than the VCD at day 7. Extended culture VCD CDCLs were shown to maintain a high VCD between days 7–10. This could be attributed to a longer stationary phase than normal culture VCD CDCLs or perhaps later peak VCD. The CDCLs chosen for this experiment were different to those chosen for high/low peak VCD and were analysed separately; however, it should be noted that there was overlap in the CDCLs chosen for low peak VCD and normal culture VCD as seen in Fig. 1. The main difference between high/low peak VCD CDCLs and extended/normal VCD CDCLs is that extended culture VCD have a prolonged stationary phase. Extended culture VCD CDCLs, do not reach as high a VCD as high peak VCD CDCLs, with a peak VCD of 12.3 × 10^6^ cells/mL for high peak VCD CDCLs and 10.4 × 10^6^ cells/mL for extended culture VCD CDCLs. Day 6 represents the exponential growth phase of these CDCLs. Day 10 represents the early decline phase of growth for normal culture VCD CDCLs and the prolonged stationary phase of growth for extended culture VCD CDCLs. Extended culture VCD CDCLs exhibited a significantly higher VCD at the later time-points when compared to normal culture VCD CDCLs (Fig. 3). TCD was also found to be significantly higher in extended culture VCD CDCLs at day 10 and IVCD was found to be higher in extended VCD CDCLs at day 7 and 10 (Fig. 3, Additional files 5 and 6). No significant difference between extended and normal culture VCD CDCLs was detected in viability, titre, Qp, cell size, cell volume, gene copy number or transcript copy number (Fig. 3 and Additional file 4). No significant difference in lactate or ammonia, glucose or glutamate levels were detected between normal and extened culture VCD CDCLs (Additional file 4). Glutamine levels were found to be significantly higher in normal culture VCD CDCLs at day 14.

### Differential LC-MS/MS proteomic analysis of growth phenotypes in CHO cell CDCLs

#### High/low peak VCD CDCLs

Over 4000 proteins were consistently identified in each high and low peak VCD sample using LC-MS/MS analysis on the Orbitrap Fusion Tribrid MS in a 90 min LC-MS run for each sample. High and low peak VCD CDCLs were analysed using differential LC-MS/MS analysis and 229 DE proteins were identified at day 6 (Additional file 1). Of the proteins identified, 128 were found to have increased expression and 101 proteins were found to have decreased expression in the high peak VCD CDCLs. At day 10,182 DE proteins were identified (Additional file 1), 85 of which were found to have increased expression and 97 with decreased expression in high peak VCD CDCLs. We identified 36 proteins which were DE between high and low peak VCD CDCLs at day 6 and day 10 (Table 1). GO analysis identified an over-representation of proteins associated with RNA processing in high peak VCD CDCLs. Specifically, an enrichment of proteins associated with ribonucleoprotein (RNP) complex biogenesis was observed in high peak VCD CDCLs at day 6 (Table 1). Several RNP complex biogenesis associated proteins which were shown to have increased expression in high Qp CDCLs have also been associated with evading and regulating p53 mediated apoptosis and cell cycle arrest (e.g. DDX31, DKC1, PRMT5, NOC2L, PES1) (Table 2).

**Table 1:** Proteins DE at both day 6 and 10 between high and low peak VCD CDCLs or between normal and extended culture VCD CDCLs.

**Gene**	**Accession**	**Description**	**D6 Fold change**	**D6 Phenotype**	**D10 Fold change**	**D10 Phenotype**
MCM2	G3H7V9	DNA helicase	2.56	Up in high peak VCD	2.63	Up in high peak VCD
XPO7	G3GUX2	Exportin-7 (Fragment)	2.43	Up in high peak VCD	2.06	Up in high peak VCD
EPHA2	G3I863	Ephrin type-A receptor 2	2.37	Up in high peak VCD	1.63	Up in high peak VCD
SMC4	G3IAU0	Uncharacterized protein (Fragment)	2.28	Up in high peak VCD	2.73	Up in high peak VCD
PGM2	G3IFE7	Phosphoglucomutase-2	2.14	Up in high peak VCD	1.53	Up in high peak VCD
PDE12	G3GUS6	2′,5′-phosphodiesterase 12	2.09	Up in high peak VCD	1.73	Up in high peak VCD
SEPSECS	G3I841	O-phosphoseryl-tRNA (Sec) selenium transferase	2.05	Up in high peak VCD	1.66	Up in high peak VCD
TRIP13	G3ILQ1	Thyroid receptor-interacting protein 13	2.00	Up in high peak VCD	2.59	Up in high peak VCD
NCAPD2	G3GUM5	Condensin complex subunit 1	1.89	Up in high peak VCD	4.88	Up in high peak VCD
SF3B3	G3HAF4	Splicing factor 3B subunit 3	1.85	Up in high peak VCD	1.56	Up in high peak VCD
NCAPG	G3HR05	Condensin complex subunit 3	1.85	Up in high peak VCD	5.07	Up in high peak VCD
IPO4	G3HDD8	Importin-4	1.78	Up in high peak VCD	1.50	Up in high peak VCD
CDK1	G3HVL1	Cell division control protein 2-like	1.70	Up in high peak VCD	1.74	Up in high peak VCD
MCM5	G3IAI5	DNA helicase	1.67	Up in high peak VCD	2.59	Up in high peak VCD
RCC2	G3IJB6	Protein RCC2	1.66	Up in high peak VCD	2.08	Up in high peak VCD
ECM29	G3I5Z8	Proteasome-associated protein ECM29-like	1.64	Up in high peak VCD	1.51	Up in high peak VCD
PRPF6	G3IBT1	Pre-mRNA-processing factor 6	1.62	Up in high peak VCD	1.50	Up in high peak VCD
PRMT5	G3HRD3	Protein arginine N-methyltransferase 5	1.58	Up in high peak VCD	1.58	Up in high peak VCD
ELOVL7	G3GV15	Elongation of very long chain fatty acids protein 7	5.09	Up in low peak VCD	4.91	Up in low peak VCD
PC	G3I4L6	Pyruvate carboxylase	2.68	Up in low peak VCD	1.87	Up in low peak VCD
HMOX1	G3IAI6	Heme oxygenase	2.40	Up in low peak VCD	2.53	Up in low peak VCD
AOC3	G3I525	Amine oxidase (Fragment)	2.36	Up in low peak VCD	3.52	Up in low peak VCD
FLOT1	G3HQP7	Flotillin-1	2.15	Up in low peak VCD	1.51	Up in low peak VCD
XDH	G3I4G1	Xanthine dehydrogenase/oxidase	2.02	Up in low peak VCD	1.84	Up in low peak VCD
PSMC6	G3I6I1	26S protease regulatory subunit S10B	2.00	Up in low peak VCD	1.63	Up in low peak VCD
SQOR	G3HMD2	Sulfide:quinone oxidoreductase, mitochondrial	1.96	Up in low peak VCD	1.81	Up in low peak VCD
GPD1L	G3GV81	Glycerol-3-phosphate dehydrogenase [NAD(+)]	1.89	Up in low peak VCD	1.63	Up in low peak VCD
ACO1	G3HQZ8	Cytoplasmic aconitate hydratase	1.78	Up in low peak VCD	1.77	Up in low peak VCD
PARP3	G3H896	Poly [ADP-ribose] polymerase	1.77	Up in low peak VCD	1.82	Up in low peak VCD
VPS13C	G3H509	Vacuolar protein sorting-associated protein 13C	1.62	Up in low peak VCD	2.74	Up in low peak VCD
UBR4	G3I905	E3 ubiquitin-protein ligase UBR4	1.60	Up in low peak VCD	1.63	Up in low peak VCD
MYO18A	G3I7W5	Myosin-XVIIIa	1.60	Up in low peak VCD	1.99	Up in low peak VCD
CCT2	G3HZ42	T-complex protein 1 subunit beta	1.55	Up in low peak VCD	2.26	Up in low peak VCD
ACSF2	G3HBE7	Acyl-CoA synthetase family member 2, mitochondrial	1.55	Up in low peak VCD	1.82	Up in low peak VCD
MYO6	G3I0Q8	Myosin-VI	1.53	Up in low peak VCD	2.06	Up in low peak VCD
HACD3	G3HUX9	Very-long-chain (3R)-3-hydroxyacyl-CoA dehydratase	1.51	Up in low peak VCD	1.52	Up in low peak VCD
Gene	**Accession**	**Description**	**D6 Fold change**	**D6 Phenotype**	**D10 Fold change**	**D10 Phenotype**
FLNC	G3GZ94	Filamin-C	Infinity	Up in extended VCD	2.520228255	Up in extended VCD
CAND1	G3GY17	Cullin-associated NEDD8-dissociated protein 1	1.86816535	Up in extended VCD	1.840673521	Up in extended VCD
GBF1	G3HXV9	Golgi-specific brefeldin A-resistance guanine nucleotide exchange factor 1	1.7337689	Up in extended VCD	1.587167296	Up in extended VCD
THBS1	G3HHV4	Thrombospondin-1	6.13878563	Up in Normal VCD	51.1241988	Up in Normal VCD
AOC3	G3I525	Amine oxidase (Fragment)	2.28761761	Up in Normal VCD	2.430711805	Up in Normal VCD
HEXB	G3HXN7	Beta-hexosaminidase	2.24732962	Up in Normal VCD	2.440308152	Up in Normal VCD
DNM2	G3H6B2	Dynamin	2.01883385	Up in Normal VCD	1.537175512	Up in Normal VCD
TRPV2	G3GVL1	Transient receptor potential cation channel subfamily V member 2	2.01058317	Up in Normal VCD	1.877033504	Up in Normal VCD
NEK7	G3HM24	Serine/threonine-protein kinase Nek7	1.81795951	Up in Normal VCD	1.717212511	Up in Normal VCD
PICALM	G3HXR8	Phosphatidylinositol-binding clathrin assembly protein	1.80428032	Up in Normal VCD	1.615700185	Up in Normal VCD
GPD2	G3HT22	Glycerol-3-phosphate dehydrogenase	1.76165957	Up in Normal VCD	1.684923387	Up in Normal VCD
MYO18A	G3I7W5	Myosin-XVIIIa	1.73531125	Up in Normal VCD	2.435810461	Up in Normal VCD
TRAP1	G3I027	Heat shock protein 75 kDa, mitochondrial	1.69466205	Up in Normal VCD	4.665085424	Up in Normal VCD
EHD1	G3I6F9	EH domain-containing protein 1	1.65572091	Up in Normal VCD	3.187918972	Up in Normal VCD
PXDN	G3HBI1	Peroxidasin-like	1.55976418	Up in Normal VCD	2.637755938	Up in Normal VCD
						Up in Normal VCD

**Table 2:** Ribonucleoprotein complex biogenesis associated proteins associated proteins upregulated in high peak VCD CDCLs at day 6

Accession	Gene	Description	Anova (p)	Fold change	Phenotype
G3HKF6	BRIX1	Brix domain-containing protein 2	0.00111533	2.31	Up in high peak VCD
G3IFQ3	DDX31	Putative ATP-dependent RNA helicase DDX31	0.00354992	3.67	Up in high peak VCD
G3H5A9	DIS3	Exosome complex exonuclease RRP44	0.00050052	2.25	Up in high peak VCD
G3HV92	DKC1	H/ACA ribonucleoprotein complex subunit DKC1	0.0028815	3.05	Up in high peak VCD
G3GUQ7	EMG1	EMG1	0.01270532	1.55	Up in high peak VCD
G3HQR1	GNL1	Guanine nucleotide-binding protein-like 1 (Fragment)	0.01589585	1.66	Up in high peak VCD
G3H5M8	HEATR3	HEAT repeat-containing protein 3	0.00829343	1.74	Up in high peak VCD
G3GYY4	NAT10	RNA cytidine acetyltransferase	0.00165537	1.62	Up in high peak VCD
G3IJ36	NOC2L	Nucleolar complex protein 2-like	0.00044883	1.70	Up in high peak VCD
G3H451	NOP56	Nucleolar protein 56	0.00050795	2.26	Up in high peak VCD
G3IB34	PDCD11	Protein RRP5-like	0.00457232	3.07	Up in high peak VCD
G3I150	pes1	Pescadillo-like	0.00026516	1.77	Up in high peak VCD
G3HRD3	PRMT5	Protein arginine N-methyltransferase 5	0.00403556	1.58	Up in high peak VCD
G3IBT1	PRPF6	Pre-mRNA-processing factor 6	0.01633509	1.62	Up in high peak VCD
G3IPH0	PRPF8	Pre-mRNA-processing-splicing factor 8	0.02362991	1.97	Up in high peak VCD
G3HZV0	RPL10A	Ribosomal protein	0.00067857	3.74	Up in high peak VCD
G3HL16	SF3B1	Splicing factor 3B subunit 1	0.00592115	2.12	Up in high peak VCD
G3HHX7	SKIV2L2	Superkiller viralicidic activity 2-like 2	0.01145581	1.59	Up in high peak VCD
G3H916	WDR3	WD repeat-containing protein 3	0.00293993	1.81	Up in high peak VCD

Several cell cycle associated proteins were identified with increased expression in high peak VCD CDCLs at both time-points (Table 3). Increased expression of cell cycle associated proteins was found to be more significant at day 10. Many cell cycle proteins identified are specifically associated with the G1/S transition (Table 4) (e.g. CDK1, BCAT1, RPA1 DHRF, PPAT, PCNA). Proteins associated with DNA replication were also identified as having increased expression in high peak VCD CDCLs at day 10 (Table 5). Several DNA replication associated proteins identified were found to be members of the minichromosome maintenance (MCM) complex (e.g. MCM2, MCM3, MCM4, MCM5, MCM6, PCNA). All MCM complex members identified as upregulated in high peak VCD CDCLs at day 10 were found to have a similar fold changes. MCM2 and MCM5 were the only MCM proteins which was also found to have increased expression in high peak VCD CDCLs at day 6. Proteins associated with chromosome condensation were also identified as having increased expression in high peak VCD CDCLs at both time-points. Specifically, several subunits of Condensin I were found to have increased expression in high peak VCD CDCLs at day 10 (e.g. SMC2, SMC4, NCAPD2, NCAPG) (Table 6). NCAPD2 and NCAPG, were found to be more highly expressed in high peak VCD CDCLs at day 10.

**Table 3:** Cell cycle associated protein DE at day 6 and/or 10 between high and low peak VCD CDCLs

Accession	Gene	Description	Fold Change D6	Phenotype D6	Fold Change D10	Phenotype D10
G3HVL1	CDK1	Cell division control protein 2-like	1.70	Up in high VCD	1.74	Up in high peak VCD
G3GXH4	CDK6	Cell division protein kinase 6	3.47	Up in high VCD		
G3GV30	EML4	Echinoderm microtubule-associated protein-like 4 (Fragment)	1.80	Up in high VCD		
G3H7V9	MCM2	DNA helicase	2.56	Up in high VCD	2.63	Up in high peak VCD
G3IAI5	MCM5	DNA helicase	1.67	Up in high VCD	2.59	Up in high peak VCD
G3H5Q3	MSH2	DNA mismatch repair protein	2.61	Up in high VCD		
G3H5Q5	MSH6	DNA mismatch repair protein	2.50	Up in high VCD		
G3GUM5	NCAPD2	Condensin complex subunit 1	1.89	Up in high VCD	4.88	Up in high peak VCD
G3HR05	NCAPG	Condensin complex subunit 3	1.85	Up in high VCD	5.07	Up in high peak VCD
G3IBF6	PDS5A	Sister chromatid cohesion protein PDS5-like A	1.80	Up in high VCD		
G3HYF3	POLA1	DNA polymerase	1.95	Up in high VCD		
G3I732	POLD1	DNA polymerase	2.71	Up in high VCD		
G3HRM7	PRIM1	DNA primase	3.07	Up in high VCD		
G3GUU6	PRKCD	Non-specific serine/threonine protein kinase	1.62	Up in high VCD		
G3IJB6	RCC2	Protein RCC2	1.66	Up in high VCD	2.08	Up in high peak VCD
G3IAU0	SMC4	Uncharacterized protein (Fragment)	2.28	Up in high VCD	2.73	Up in high peak VCD
G3HJS1	SPTBN1	Spectrin beta chain, brain 1	1.72	Up in high VCD		
G3I2J5	SUN2	Protein unc-84-like B	1.84	Up in high VCD		
G3HMC7	TBRG4	Protein TBRG4	2.58	Up in high VCD		
G3ILQ1	TRIP13	Thyroid receptor-interacting protein 13	2.00	Up in high VCD		
G3HK64	TUBGCP3	Gamma-tubulin complex component	3.03	Up in high VCD		
G3HHD8	VRK1	Serine/threonine-protein kinase VRK1	1.69	Up in high VCD		
G3GXR7	MAPRE3	Microtubule-associated protein RP/EB family member 3	1.99	Up in low peak VCD		
G3HYW2	MCTS1	Malignant T cell amplified sequence 1	1.77	Up in low peak VCD		
G3H2N6	PDCD6IP	Programmed cell death 6-interacting protein	2.40	Up in low peak VCD		
G3H3D3	BCAT1	Branched-chain-amino-acid aminotransferase			87.94	Up in high peak VCD
G3I5V6	BUB3	Mitotic checkpoint protein BUB3			2.16	Up in high peak VCD
G3GUY5	CCAR2	Protein KIAA1967-like			1.77	Up in high peak VCD
Q2MH30	DHFR	Dihydrofolate reductase			3.44	Up in high peak VCD
G3H9Z5	EPS8	Epidermal growth factor receptor kinase substrate 8			1.85	Up in high peak VCD
G3H354	HSP90AA1	Heat shock protein HSP 90-alpha			1.52	Up in high peak VCD
						Up in high peak VCD
G3I1H0	MCM3	DNA helicase			2.75	Up in high peak VCD
G3I2I1	MCM4	DNA helicase			2.42	Up in high peak VCD
G3IAI5	MCM5	DNA helicase			2.59	Up in high peak VCD
G3GZQ9	MCM6	DNA helicase			2.57	Up in high peak VCD
G3IFZ0	MKI67	Antigen KI-67			24.45	Up in high peak VCD
G3HR05	NCAPG	Condensin complex subunit 3			5.07	Up in high peak VCD
G3HC95	NSUN2	tRNA (Cytosine-5-)-methyltransferase NSUN2			1.56	Up in high peak VCD
G3H412	PCNA	Proliferating cell nuclear antigen			2.72	Up in high peak VCD
G3IFL1	PPAT	Amidophosphoribosyltransferase			2.07	Up in high peak VCD
G3IP86	RPA1	Replication protein A 70 kDa DNA-binding subunit			1.52	Up in high peak VCD
G3IDS7	SLC16A1	Monocarboxylate transporter 1			1.64	Up in high peak VCD
G3GTY6	SMC2	Structural maintenance of chromosomes protein 2			5.64	Up in high peak VCD
G3IAU0	SMC4	Uncharacterized protein (Fragment)			2.73	Up in high peak VCD
G3I5N5	TOP2A	DNA topoisomerase 2			10.44	Up in high peak VCD
G3ILQ1	TRIP13	Thyroid receptor-interacting protein 13			2.59	Up in high peak VCD
G3HLY0	TUBG1	Tubulin gamma chain			2.15	Up in high peak VCD
G3IFY1	TYMS	TYMS			2.59	Up in high peak VCD
G3HT32	CUL2	CUL2			1.71	Up in high peak VCD
G3HM24	NEK7	Serine/threonine-protein kinase Nek7			1.73	Up in high peak VCD
G3GV75	RALA	RALA			1.50	Up in high peak VCD

**Table 4:** G1/S transition of mitotic cell cycle associated protein which are upregulated high peak VCD CDCLs at day 10

Accession	Gene	Description	Anova	Fold Change	Phenotype
G3H3D3	BCAT1	Branched-chain-amino-acid aminotransferase	0.04461735	87.94	Up in high peak VCD D10
G3HVL1	CDK1	Cyclin-dependent kinase 1	0.0046353	1.74	Up in high peak VCD D10
Q2MH30	DHFR	Dihydrofolate reductase	0.00099149	3.44	Up in high peak VCD D10
G3H7V9	MCM2	DNA replication licensing factor MCM2	0.00207961	2.63	Up in high peak VCD D10
G3I1H0	MCM3	DNA replication licensing factor MCM3	0.01451645	2.75	Up in high peak VCD D10
G3I2I1	MCM4	DNA replication licensing factor MCM4	0.00244631	2.42	Up in high peak VCD D10
G3IAI5	MCM5	DNA replication licensing factor MCM5	0.00522577	2.59	Up in high peak VCD D10
G3GZQ9	MCM6	DNA replication licensing factor MCM6	0.00037305	2.57	Up in high peak VCD D10
G3IFL1	PPAT	Amidophosphoribosyltransferase	0.00210528	2.07	Up in high peak VCD D10
G3H412	PCNA	Proliferating cell nuclear antigen	0.01155428	2.72	Up in high peak VCD D10
G3IP86	RPA1	Replication protein A 70 kDa DNA-binding subunit	0.01339932	1.52	Up in high peak VCD D10
G3IFY1	TYMS	TYMS	0.00011434	2.59	Up in high peak VCD D10

**Table 5:** DNA replication associated protein which are upregulated in high/low peak VCD CDCLs at day 10

Accession	Gene	Description	Anova	Fold Change	Phenotype
G3HWP7	SUPT16H	FACT complex subunit SPT16	0.00641599	1.59	Up in high peak VCD D10
G3HVL1	CDK1	Cyclin-dependent kinase 1	0.0046353	1.74	Up in high peak VCD D10
G3H7V9	MCM2	DNA replication licensing factor MCM2	0.00207961	2.63	Up in high peak VCD D10
G3I1H0	MCM3	DNA replication licensing factor MCM3	0.01451645	2.75	Up in high peak VCD D10
G3I2I1	MCM4	DNA replication licensing factor MCM4	0.00244631	2.42	Up in high peak VCD D10
G3IAI5	MCM5	DNA replication licensing factor MCM5	0.00522577	2.59	Up in high peak VCD D10
G3GZQ9	MCM6	DNA replication licensing factor MCM6	0.00037305	2.57	Up in high peak VCD D10
G3H412	PCNA	Proliferating cell nuclear antigen	0.01155428	2.72	Up in high peak VCD D10
G3IP86	RPA1	Replication protein A 70 kDa DNA-binding subunit	0.01339932	1.52	Up in high peak VCD D10

**Table 6:** Chromosome condensation associated proteins DE at day 6 and/or day 10 in high/low peak VCD CDCLs

Accession	Gene	Description	D6 Fold Change	D6 Phenotype	D10 Fold Change	D10 Phenotype
G3GUM5	NCAPD2	Condensin complex subunit 1	1.89	Up in high peak VCD D6	4.88	Up in high peak VCD D10
G3HR05	NCAPG	Condensin complex subunit 3	1.85	Up in high peak VCD D6	5.07	Up in high peak VCD D10
G3IAU0	SMC4	Structural maintenance of chromosomes protein 4	2.28	Up in high peak VCD D6	2.73	Up in high peak VCD D10
G3GTY6	SMC2	Structural maintenance of chromosomes protein 2			5.64	Up in high peak VCD D10

#### Extended/Normal culture VCD

Over 4000 proteins were consistently identified in each extended and normal culture VCD sample using LC-MS/MS analysis on the Orbitrap Fusion Tribrid MS in a 90 min LC-MS run for each sample. Differential LC-MS/MS analysis identified 52 proteins which were DE between extended and normal culture VCD CDCLs at day 6 (Additional file 1). We identified 11 proteins with increased expression and 41 proteins with decreased expression in extended culture VCD CDCLs. At day 10 99 DE proteins were identified (Additional file 1), 43 of which were increased and 56 were decreased in extended culture VCD CDCLs. 16 proteins were found to be DE at both time-points (Table 1). Using GO analysis we identified increased expression of proteins associated with ER to Golgi vesicle mediated transport in extended culture VCD CDCLs at day 10 of culture (e.g. SEC24C, GOLGB1, USO1, ARCN1, GBF1) (Table 7). Several proteins associated with response to stress were identified as having decreased expression in extended culture VCD CDCLs at day 10 (Table 8). Proteins specifically associated with endocytosis were identified as having decreased expression in extended culture VCD CDCLs at both time-points (Table 9). A number of these proteins have been found to be involved in the response to oxidative stress (e.g. MAOA, ICAM1, MAPK1, PXDN, TRAP1).

**Table 7:** ER to Golgi vesicle mediated transport associated proteins found to be up in high extended VCD CDCLs at day 10

Accession	Gene	Description	Anova	Fold change	Phenotype
G3GWP1	SEC24C	Protein transport protein Sec24C	0.00139168	1.52	Up in extended VCD Day 10
G3I9F0	USO1	General vesicular transport factor p115	0.00584826	1.95	Up in extended VCD Day 10
G3I5S7	ARCN1	Coatomer subunit delta	0.03673471	1.52	Up in extended VCD Day 10
G3HXV9	GBF1	Golgi-specific brefeldin A-resistance guanine nucleotide exchange factor 1	0.00265527	1.59	Up in extended VCD Day 10
G3HAJ0	GOLGB1	Golgin subfamily B member 1	0.01562743	1.85	Up in extended VCD Day 10

**Table 8:** Stress response associated proteins upregulated in normal VCD CDCLs at day 10

Accession	Gene	Description	Anova	Fold Change	Phenotype
G3I525	MAOA	Amine oxidase [flavin-containing] A	1.06458E-06	2.43	Up in normal VCD
G3HMG4	APP	Amyloid beta A4 protein	0.027319884	2.00	Up in normal VCD
G3H8H7	DNAJC10	DnaJ homolog subfamily C member 10	0.006170395	11.49	Up in normal VCD
G3H6B2	DNM2	Dynamin	0.002401573	1.54	Up in normal VCD
G3I4G0	EHD1	EH domain-containing protein 3	0.029785452	3.19	Up in normal VCD
G3HQP7	FLOT1	Flotillin-1	0.024002492	1.60	Up in normal VCD
G3I1V3	FN1	Fibronectin	0.003066934	1230.93	Up in normal VCD
Q9ERF7	ICAM1	Intercellular adhesion molecule 1	0.005818192	1.65	Up in normal VCD
G3H3P5	KIF5B	Kinesin-like protein	0.002186579	1.57	Up in normal VCD
G3H6V7	LPL	Lipoprotein lipase	0.006669695	2.47	Up in normal VCD
G3I4H1	MAPK1	Mitogen-activated protein kinase	0.008512686	1.58	Up in normal VCD
G3GUV3	MMP12	Macrophage metalloelastase	0.028820981	3.67	Up in normal VCD
G3H5N7	MRPS9	28S ribosomal protein S9, mitochondrial	0.009356398	2.48	Up in normal VCD
G3I0Q8	MYO6	Myosin-VI	0.028110309	1.73	Up in normal VCD
G3HQV2	OXSR1	Serine/threonine-protein kinase OSR1	0.00603464	1.92	Up in normal VCD
G3HBI1	PXDN	Peroxidasin-like	0.032863964	2.64	Up in normal VCD
G3HYQ6	RPS6KA1	Ribosomal protein S6 kinase	0.00520172	1.85	Up in normal VCD
G3HLT0	SLPI	Antileukoproteinase	0.032380155	3.55	Up in normal VCD
G3HHV4	THBS1	Thrombospondin-1	0.009824159	51.12	Up in normal VCD
G3I027	TRAP1	Heat shock protein 75 kDa, mitochondrial	0.028863637	4.67	Up in normal VCD
G3GVL1	TRPV2	Transient receptor potential cation channel subfamily V member 2	0.009029784	1.88	Up in normal VCD

**Table 9:** Endocytosis associated proteins found to be upregulated in normal VCD CDCLs at day 6 and/or day 10

Accession	Gene	Description	Fold Change D6	Phenotype D6	Fold Change D10	Phenotype D10
G3I319	AP2M1	AP-2 complex subunit mu-1			1.60	Up in normal VCD
G3HMG4	APP	Amyloid beta A4 protein			2.00	Up in normal VCD
G3HI96	CORO1C	Coronin			1.53	Up in normal VCD
G3H6B2	DNM2	Dynamin	2.02	Up in normal VCD	1.54	Up in normal VCD
G3I4G0	EHD1	EH domain-containing protein 3	1.66	Up in normal VCD	3.19	Up in normal VCD
G3I4H1	MAPK1	Mitogen-activated protein kinase			1.58	Up in normal VCD
G3I0Q8	MYO6	Myosin-VI			1.73	Up in normal VCD
G3HXR8	PICALM	Phosphatidylinositol-binding clathrin assembly protein	1.80	Up in normal VCD	1.62	Up in normal VCD
G3HHV4	THBS1	Thrombospondin-1			51.12	Up in normal VCD
G3ICV1	INPPL1	Phosphatidylinositol-3,4,5-trisphosphate 5-phosphatase 2	1.90	Up in normal VCD		
G3GVF5	SNX8	Sorting nexin-8	4.09	Up in normal VCD		

### Overlap in DE proteins identified in each experiments

In this study we investigated two growth related phenotypes “High/low peak VCD” and “Extended/Normal culture VCD”. Differentially expressed proteins associated with each phenotypes were identified. We identified 42 proteins which were found to be DE in both experiments (Table 10). For the majority of these proteins (39 of 42), high peak VCD was found to correlate with extended culture VCD and low peak VCD was found to correlate with normal culture VCD. GO analysis was performed on the list of overlapping proteins between both experiments; however, no particularly strong pathway enrichment was detected. A number of proteins associated with the cell cycle were identified as DE in both experiments (e.g. MCM3, NEK7, SUN2 and HSP90AB1).

**Table 10:** Proteins which were identified as differentially expressed in both experiments

Accession	Gene	Description	High/low peak VCD	Normal/ Extended VCD	High/low peak VCD Fold Change	Normal/ Extended VCD Fold change
G3I4X8	SBNO1	Protein strawberry notch-like 2	Up in high peak VCD D6	Up in normal VCD D6	8.84	1.58
G3I525	AOC3	Amine oxidase (Fragment)	Up in high peak VCD D6	Up in normal VCD D6	2.36	2.29
G3HUX4	USP14	Ubiquitin carboxyl-terminal hydrolase 14	Up in high peak VCD D6	Up in normal VCD D6	1.78	2.65
G3HT22	GPD2	Glycerol-3-phosphate dehydrogenase	Up in high peak VCD D6	Up in normal VCD D6	1.69	1.76
G3I7W5	MYO18A	Myosin-XVIIIa	Up in high peak VCD D6	Up in normal VCD D6	1.60	1.74
G3IMX9	VWA5A	von Willebrand factor A domain-containing protein 5A	Up in high peak VCD D6	Up in normal VCD D6	1.56	2.25
G3HUM5	GPI	SUMO-activating enzyme subunit 2	Up in high peak VCD D10	Up in Extended VCD D10	1.70	2.41
G3I1H0	MCM3	DNA helicase	Up in high peak VCD D10	Up in Extended VCD D10	2.75	1.59
G3HM24	NEK7	Serine/threonine-protein kinase Nek7	Up in high peak VCD D10	Up in normal VCD D10	1.73	1.82
G3I8U4	SMCHD1	EMILIN-2 (Fragment)	Up in high peak VCD D6	Up in Extended VCD D6	5.16	1.52
G3HCY7	TRMT1	tRNA (guanine(26)-N(2))-dimethyltransferase	Up in high peak VCD D6	Up in Extended VCD D6	3.09	1.88
G3GRE1	EPRS	Bifunctional aminoacyl-tRNA synthetase (Fragment)	Up in high peak VCD D6	Up in Extended VCD D6	1.91	1.54
G3I2J5	SUN2	Protein unc-84-like B	Up in high peak VCD D6	Up in Extended VCD D6	1.84	1.51
G3INF7	GSTM1	Glutathione S-transferase Mu 1	Up in high peak VCD D6	Up in Extended VCD D6	1.67	1.90
G3H7T9	HUWE1	E3 ubiquitin-protein ligase HUWE1 (Fragment)	Up in high peak VCD D6	Up in Extended VCD D10	2.38	2.22
G3ILF9	PYGL	Alpha-1,4 glucan phosphorylase	Up in high peak VCD D6	Up in Extended VCD D10	2.35	2.32
G3GUS6	PDE12	2′,5′-phosphodiesterase 12	Up in high peak VCD D6	Up in Extended VCD D10	2.09	1.91
G3GXT2	CAD	CAD protein	Up in high peak VCD D6	Up in Extended VCD D10	1.94	1.56
G3I7H7	ACAD9	Acyl-CoA dehydrogenase family member 9, mitochondrial	Up in high peak VCD D6	Up in Extended VCD D10	1.79	1.52
G3HDD8	IPO4	Importin-4	Up in high peak VCD D6	Up in Extended VCD D10	1.78	1.60
G3HYU7	XPO5	Exportin-5	Up in high peak VCD D6	Up in Extended VCD D10	1.66	1.97
G3HRD3	PRMT5	Protein arginine N-methyltransferase 5	Up in high peak VCD D6	Up in Extended VCD D10	1.58	1.74
G3HQP7	FLOT1	Flotillin-1	Up in low peak VCD D10	Up in normal VCD D10	2.15	1.60
G3HKN0	IGF2BP2	Insulin-like growth factor 2 mRNA-binding protein 3	Up in low peak VCD D10	Up in normal VCD D10	1.92	2.38
G3H3P5	KIF5B	Kinesin-like protein	Up in low peak VCD D10	Up in normal VCD D10	1.82	1.57
G3HQZ8	ACO1	Cytoplasmic aconitate hydratase	Up in low peak VCD D10	Up in normal VCD D10	1.78	6.75
G3H3G9	SEPTIN9	Septin-9	Up in low peak VCD D10	Up in Extended VCD D10	1.72	1.63
G3HBE7	ACSF2	Acyl-CoA synthetase family member 2, mitochondrial	Up in low peak VCD D10	Up in normal VCD D10	1.55	1.60
G3I0Q8	MYO6	Myosin-VI	Up in low peak VCD D10	Up in normal VCD D10	1.53	1.73
G3H3I2	IPO9	Importin-9	Up in high peak VCD D10	Up in Extended VCD D10	1.54	1.73
G3HQM6	HSP90AB1	Endoplasmin	Up in high peak VCD D10	Up in Extended VCD D10	1.67	3.56
G3GUY5	CCAR2	Protein KIAA1967-like	Up in high peak VCD D10	Up in Extended VCD D10	1.77	1.82
G3H1D2	COIL	Tripartite motif-containing protein 25	Up in low peak VCD D10	Up in normal VCD D10	1.67	1.65
G3GSJ7	BDH1	BDH1	Up in low peak VCD D10	Up in normal VCD D10	1.75	2.02
G3GRY1	HSDL1	HSDL1	Up in low peak VCD D10	Up in normal VCD D10	1.77	1.70
G3I0F7	NAGA	Alpha-galactosidase	Up in low peak VCD D10	Up in normal VCD D10	1.78	1.75
G3IBR4	SEPTIN3	Neuronal-specific septin-3	Up in low peak VCD D10	Up in normal VCD D10	2.02	2.08
G3HTA6	EPM2AIP1	EPM2A-interacting protein 1	Up in low peak VCD D10	Up in Extended VCD D10	2.08	2.18
G3I6D1	HSD3B1	3 beta-hydroxysteroid dehydrogenase/Delta 5-->	Up in low peak VCD D10	Up in normal VCD D10	2.90	1.74
G3H3Y2	GAG	Retrovirus-related Gag polyprotein	Up in low peak VCD D10	Up in normal VCD D10	3.24	1.53
G3GUV3	MMP12	Macrophage metalloelastase	Up in low peak VCD D10	Up in normal VCD D10	4.34	3.67
G3HLT0	SLPI	Antileukoproteinase	Up in low peak VCD D10	Up in normal VCD D10	5.43	3.55

## Discussion

**Fig. 4:**
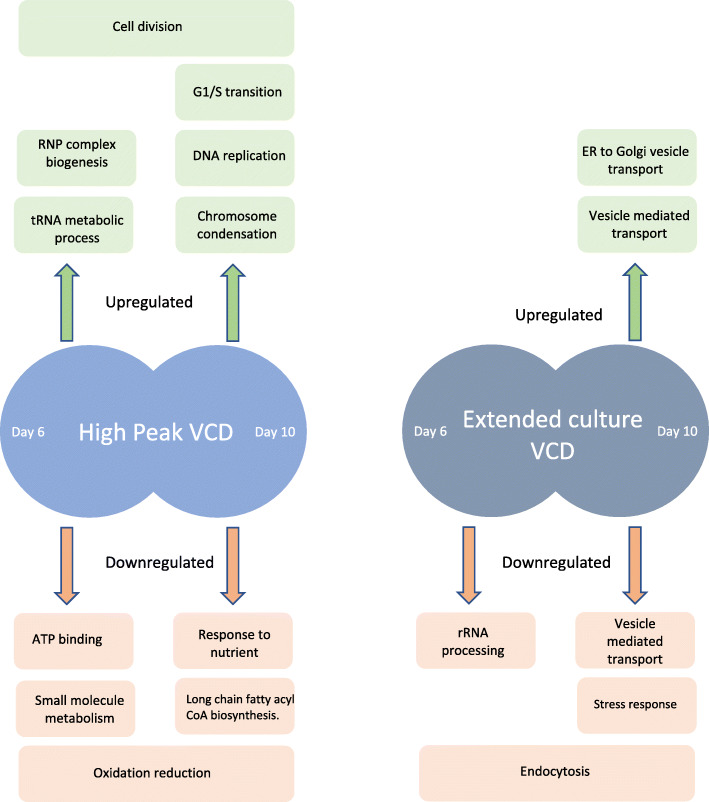
Schematic representation of biological processes upregulated and downregulated in (A) high/low peak VCD CDCLs (B) high extended VCD CDCLs

The aim of this study was to improve our understanding of the molecular basis for desirable growth phenotypes in industrially relevant CHO CDCLs. Engineering CHO cell lines with increased VCD and extended culture VCD has the potential to help maintain high Qp and titre output. The correlation between growth and Qp in CHO cells has been well demonstrated, with maximum productivity usually being observed in the stationary phase [42, 43]. For this study, high/low peak VCD CDCLs and normal/extended VCD CDCLs were found to have no statistically significant differences in titre or Qp. However, the trend observed was for high peak VCD CDCLs to have a lower titre and Qp, and for extended VCD CDCLs to have a higher titre and Qp. These observations suggest that in order to optimise recombinant protein production in CHO cells an extended culture VCD is required in addition to a high peak VCD in order to achieve high titre and Qp. Achieving high peak VCD quickly in culture has the potential to decrease culture process length and in turn potentially reduce costs associated with production. Intensified fed batch is a system which has been described in many recent studies (Jordan et al. 2018; Yongky et al. 2019; Xu et al. 2020). This system has been successfully applied to reach the peak VCD earlier by seeding the production stage at a much higher density, therefore hitting peak VCD earlier on and shortening the duration of the cell culture. The high peak VCD CDCL phenotype described in this study could mimic the intensified fed batch process without the need for high seed density while lowering the cost of production (COPS) due to shorter process duration. Extending culture viability has previously been shown to improve Qp where mitochondrial dysfunction inhibitors, Bcl-X(L) and Aven, and a caspase inhibitor of cell death were used to reduce apoptosis in culture [44]. The ability to create an extended high culture VCD can also simplify downstream processing steps by reducing host cell impurities resulting from lysed dead cells and ultimately reducing costs associated with downstream processing. Bioprocess parameters such as temperature and media formulation have been shown to have limited impact on CHO host cell impurity profiles [45] [45–47].. The creation of an extended high culture VCD would help reduce cell death in culture and in turn reduce host cell impurities which must be removed during downstream processing. Interestingly, although both phenotypes investigated in this study were related to cell growth, they highlighted unique biological processes, with little overlap in DE proteins between experiments, suggesting that in order to engineer a high peak VCD / extended culture VCD CHO cell line multiple proteins/pathways would need to be targeted. Figure 4 illustrates biological processes which were significantly DE in each experimental group. We also observed a trend of higher gene copy numbers in high peak VCD and extended VCD CDCLs. however, this was not found to be statistically significant due to a high level of variance in gene copy number between high VCD CDCLs. Variation in transgene copy number observed in the CHO genome occurs as a result of random integration of expression vectors into multiple different genomic loci (Grav et al., 2018).

### High peak VCD phenotype

When investigating the high peak VCD phenotype, we observed that several RNP complex biogenesis associated proteins identified with increased expression in the high peak VCD CDCLs have been associated with evading and regulating p53 mediated apoptosis and cell cycle arrest (e.g. DDX31, DKC1, PRMT5, NOC2L, PES1). Downstream targets of p53 have been shown to regulate pathways such as apoptosis, cell cycle arrest and DNA repair. P53 is usually activated by cell stress such as hypoxia, DNA damage and lack of nutrients or growth factors [48–50]. Putative ATP-dependent RNA helicase DDX31 (DDX31) was found to have 3.67 fold increased expression in high peak VCD CDCLs. DDX31 is understood to regulate the p53-MDM2 pathway by binding nucleophosmin (NPM) and inhibiting NPM interacting with MDM2 [51]. If NPM cannot bind to p53, then p53 induced apoptosis and cell cycle arrest is also inhibited. H/ACA ribonucleoprotein complex subunit DKC1 (DKC1) represents another example of proteins which are involved in evading and regulating p53 mediated apoptosis and cell cycle arrest. DKC1 was found in this study to have 3.05 fold increased expression in high peak VCD CDCLs. Studies have shown that mutation in DKC1 in mice results in slow growth due to increased DNA damage via the ATM/p53 pathway [52]. Results of this study show a clear enrichment of RNP complex biogenesis proteins associated with evading P53 mediated apoptosis and/or cell cycle arrest in response to intrinsic and extrinsic stress signals in CDCLs displaying a high peak VCD phenotype at day 6. At this time-point, CDCLs are in the exponential phase of growth. This is when we observe the greatest difference in VCD between high and low peak VCD CDCLs. Pathways functioning at this time-point would be expected to have the greatest influence on growth rate and VCD. These results could suggest the ability of CDCLs which reach high peak VCDs to evade P53 mediated apoptosis, and cell cycle arrest allows them to grow faster and reach a higher VCD than low peak VCD CDCLs.

The most significant enrichment of cell cycle associated proteins were identified at day 10 (Table 3). This is an interesting observation given the fact that at day 10 the VCD of both high and low peak VCD CDCLs have begun to decline. This could suggest that high VCD CDCLs are attempting to maintain high levels cell proliferation beyond day 10 and that the reduction in VCD observed at day 10 is caused by other factors such as reduced response to nutrients. However, it should also be considered that the enrichment of cell cycle associated protein would likely still be evident at day 10 if expression of proteins decreased in both phenotypes as VCD decreases in each phenotype. A number of proteins associated with G1/S transition and the S/G2 phase of the mitotic cell cycle were found to have increased expression in high peak VCD CDCLs at day 10. Transitioning from the G1 phase of the cell cycle to the S phase is extremely important for cell proliferation [53]. It is the responsibility of cyclin dependent kinases (CDKs) to promote DNA replication and also cause G1/S phase transition [53]. In this study, cyclin-dependent kinase 1 (CDK1) was found to have 1.7 fold increased expression in high peak VCD CDCLs at day 6 and 1.74 fold increased expression in high peak VCD CDCLs at day 10. Branched-chain-amino-acid aminotransferase (BCAT1) was found to have 87.94 fold increased expression high peak VCD CDCLs at day 10. Studies of BCAT1 in yeast have suggested a role for this protein in regulating G1 to S transition [54].

Several members of the mini-chromosome maintenance (MCM) complex were also found to have increased expression in high peak VCD CDCLs (Table 4). The MCM complex controls DNA replication during the cell cycle in eukaryotic cells and can both unwind duplex DNA and is responsible for initiating fork progression [55]. There are 6 MCM proteins which comprise the hexameric ring which is found in the replicative helicase. In this study, we identified 5 out of 6 members of the MCM complex as upregulated in high peak VCD CDCLs at day 10. The similar levels of expression of each member of the MCM complexes in high peak VCD CDCLs at day 10 could suggest higher levels of DNA replication are occurring in high peak VCD CDCLs at day 10 but not day 6.

Proteins associated with chromosome condensation were also found to have increased expression in high peak VCD CDCLs (Table 5). Chromosome compaction is an essential step for genome segregation during mitosis [56]. It is the role of Condensin I and II complexes to mediate chromosome compaction. The condensin I complex is made up of structural maintenance of chromosomes 2 (SMC2) and SMC4, condensin complex subunit 1 (NCAPD2), Condensin complex subunit 3 (NCAPG) and Condensin complex subunit 2 (NCAPH) [57, 58]. Condensin I complex subunits were observed as being more significantly DE at day 10. At day 10 VCD has begun to decline significantly. The high fold changes observed in NCAPG and NCAPD2 between high and low peak VCD CDCLs at day 10 could represent a significant drop in expression of these proteins in low peak VCD CDCLs when cell death begins to increase. Whereas high peak VCD CDCLs may continue to stimulate expression of the condensin I complex and thus mitosis.

### Normal/ extended culture VCD phenotype

A high peak VCD and fast growth rate are highly desirable phenotypes in CHO cell lines producing therapeutic proteins. However, these fast growing CDCLs can quickly reach high VCDs but often only maintain these densities for a short period of time before decreasing rapidly for the remainder of the cell culture. For this reason, we believed it would also be very important to map the molecular basis for extended culture VCD in CHO CDCLs. Extended VCD CDCLs have a prolonged stationary phase. This phenotype is desirable as it allows CDCLs to maintain a high VCD for longer in culture. Often in order to achieve an extended culture VCD phenotype growth rate and peak VCD will be compromised. Here we attempt to map the molecular basis for both of these phenotypes in order to better understand their similarities and differences.

A number of proteins associated with endoplasmic reticulum (ER) to Golgi vesicle mediated transport were found to have increased expression in extended culture VCD CDCLs (Table 7). Protein transport protein Sec24C (SEC24C) was found to have a 1.52 fold increased expression in cells with extended culture VCD at day 10. SEC24C is a component of the COPII complex and is mainly important for recruitment of protein cargo into the budding vesicles [59]. General vesicular transport factor p115 (USO1) was found to have a 1.95 fold increased expression in CDCLs displaying the extended culture VCD phenotype. USO1 in yeast has been shown to be essential for tethering of vesicles in ER to Golgi transport [60]. Golgin subfamily B member 1 (GOLGB1) was also found to have increased expression in extended culture VCD CDCLs by 1.85 fold. Studies have shown GOLGB1 to interact with USO1 in both yeast and mammalian cells [61–63]. These results suggest higher levels of vesicular trafficking are present in CDCLs displaying extended culture VCD. Extended culture VCD CDCLs are in the stationary phase of growth at day 10. During the stationary phase of growth, higher levels of recombinant protein are being produced which likely explains high levels of vesicular trafficking in extended culture VCD CDCLs at day 10 but not at day 6.

At day 10, the normal culture VCD CDCLs have begun to see decreased VCD and therefore increased cell death. The increased level of endocytosis observed in normal culture VCD could be a result of increased cell death signalling in normal culture VCD CDCLs at day 10. Recent studies have shown a role for endocytic trafficking in regulating levels of cell death receptors [64]. A number of proteins identified as having decreased expression in extended culture VCD CDCLs were specifically involved in the response to oxidative stress. Damage caused by oxidative stress can result in apoptosis [65]. Intercellular adhesion molecule 1 (ICAM1) was found in this study to have 1.65 fold decreased expression in extended culture VCD CDCLs at day 10. Studies have shown levels of ICAM1 are often increased in response to oxidative stress [66]. ICAM1 has also been shown to affect cell aggregation in CHO cells, with ICAM1 knockout resulting in significantly less cell aggregation [37]. Mitogen-activated protein kinase 1 (MAPK1) was shown in this study to have 1.58 fold decreased expression in extended culture VCD CDCLs. Reactive oxygen species have been shown to be capable of activating MAPK pathways [67]. Studies in CHO cells have shown that when p38 MAPK pathways and PKA are inhibited cell proliferation is inhibited due to G1 arrest [68]. These results suggest that CDCLs displaying the extended culture VCD phenotype have lower levels of oxidative stress and in turn lower levels of oxidative stress response. Studies have shown that cell stress usually results in cell death. Apoptosis is understood to be the main cause of cell death in a bioreactor environment [69–71]. Therefore, being able to identify and monitor markers of cell stress is incredibly important. Overall, these results indicate that reduced VCD in normal culture VCD CDCLs from day 10 onwards may be caused in part by increased oxidative stress and increased endocytosis which most likely causes reduced need for ER to Golgi vesicle mediated transport.

## Conclusion

The results of this study highlight intracellular pathways which are characteristic of desirable growth phenotypes in industrially relevant CHO cell lines. In this study, we investigated two phenotypes; high peak VCD and extended culture VCD which both contribute to creating industrially desirable CHO producing cell lines. Although both phenotypes investigated are inherently related to the growth of the cell, differential LC-MS/MS proteomic analysis revealed different pathways and biological processes that are significantly enriched in each experiment. We found that RNP complex biogenesis associated proteins with emphasis on evading p53 mediated apoptosis and cell cycle arrest were highlighted as important in the early exponential growth phase of culture in CDCLs which reached a high peak VCD. We also found that proteins associated with mitotic cell cycle progression, chromosome condensation and DNA replication were highly enriched in high peak VCD CDCLs in the early lag phase of growth. In contrast to this, we found that in extended culture VCD CDCLs, ER to Golgi vesicle mediated transport was of particular importance in the stationary phase and that endocytosis and the oxidative stress response were significantly downregulated. Both phenotypes investigated in this study are extremely beneficial characteristics of producer CHO CDCLs; however, in CHO cell lines reaching a high peak VCD and maintaining it throughout culture can be difficult. Targets presented in this study could be further investigated for engineering desirable growth phenotypes in CHO producing cell lines. For example overexpression of targets such as DDX31 and DKC1 could be investigated in attempts to increase VCD of CHO cells in culture. Inducing overexpression of targets such as SEC24C and USO at later time-points in culture could be investigated in attempts to maintain a high extended culture VCD. Future proteomic profiling studies of CDCLs may also be carried out with various cell culture medias to assess the contribution of nutrient balance and availability on global cell protein regulation affecting peak cell culture VCD and extended viability. The results presented here provide a deeper understanding of the intracellular pathways which influence these growth related phenotypes in CHO cell lines.

## Methods

### Fed-batch cultivation of CHO cell lines

CHO CDCLs used in this study were generated and provided by Eli Lilly and Company. High peak VCD, low peak VCD, extended culture VCD and normal culture VCD CDCLs were seeded in E250 mL shake flasks containing 100 mL of Lilly propriety production medium at 0.75 × 10^6^ cells/mL. Cell lines were cultured at 150 rpm, 6% CO_2_ and 36 °C, with a temperature shift to 32 °C on day 4. Each CDCL was cultured in duplicate for 17 days in a Kuhner Shaker ISF1-X (Kuhner). Neutral feeds and an acidic feed were administered on days 4, 7 and 10. A glucose feed was also given on days 12 and 14 if required. Cell viability and density were measured using an automated Vicell™ XR cell viability analyzer (Beckman Coulter, Brea, CA). Specific growth rate (in reciprocal hours, h^− 1^) and cell specific productivity (Qp) was measured using the calculation described below and as previously published [72]. An outline of CDCLs used for each experiment and the proteomic experimental workflow is shown in Additional file 2.

$$ Daily\ growth\ rate=\frac{\left(\ln (density2)-\ln (density1)\right)/\left( time2- time1\right)}{24} $$

1 $$ \mathrm{Qp}\left(\mathrm{pg}/\mathrm{cell}/\mathrm{day}\right)=\left[\frac{\mathrm{titre}2-\mathrm{titre}1}{\left(\mathrm{density}2-\mathrm{density}1\right)}\right]\mathrm{x}\ \mathrm{daily}\ \mathrm{growth}\ \mathrm{rate} $$

Metabolite levels (e.g., lactate, ammonia, glutamate, glutamine and glucose) were measured on days 4, 7, 10, 14 and 17 of culture using a ABL-9000 (Radiometer America) according to manufacturer’s instructions. Gene copy numbers and transcript copy numbers of CDCLs were generated using rtqPCR with TaqMan probes and primers as previously described [73].

### Protein extraction and in-solution protein digestion

Cell pellets were taken from duplicate flasks on day 6 and day 10 of culture. On day 10, samples were taken before neutral and acidic feeds were administered. Cell pellets were harvested and washed in phosphate buffered saline. Cell pellets were lysed with lysis buffer and centrifuged at 14,000 xg for 15 min. 0.5 M dithiothreitol (DTT) was added to each lysate, which was then incubated for 20 min at 56 °C. Protein concentration was determined using Bradford assay (Bio-rad). The Filter Aided Sample Preparation (FASP) method and C18 peptide purification were then used to prepare 100 μg of each sample for LC-MS/MS analysis as described in [74]. Protein digestion was performed using a 1:200 (enzyme:protein) ratio of Lys-C (Thermo Fisher Scientific), followed by a 1:100 (enzyme:protein) ratio of sequence grade trypsin (Thermo Fisher Scientific). The basic workflow for sample preparation is illustrated in Additional file 2 (B).

### LC-MS/MS

Reverse-phased capillary high pressure liquid chromatography was used to profile total protein lysates of high/low peak VCD and normal/extended culture VCD CHO cell CDCLs. An UltiMate 3000 nano RSLC (Thermo Scientific) system interfaced with an Orbitrap Fusion Tribrid Mass Spectrometer (Thermo Scientific) was used for LC-MS/MS profiling. One microgram from each sample was loaded onto the trapping column (PepMap100, C18, 300 μm × 5 mm) at a flow rate of 25 μL/min with 2% (v/v) acetonitrile (ACN), 0.1% (v/v) trifluoroacetic acid (TFA) for 3 min. Each sample was then resolved onto an analytical column (Acclaim PepMap 100, 75 μm × 50 cm, 3 μm bead diameter column). A binary gradient of: solvent A (0.1% (v/v) formic acid in LC-MS grade water) and solvent B (80% (v/v) ACN, 0.08% (v/v) formic acid in LC-MS grade water) using 2–32% B for 75 min, 32–90% B in 5 min and holding at 90% for 5 min at a flow rate of 300 nL/min was used to elute peptides. A temperature of 320 °C and a voltage of 2.0 kV was used for peptide ionization. Data-dependent acquisition was performed using a full scan range of 380–1500 m/z. The Orbitrap mass analyser with a resolution of 120,000 (at m/z 200), a maximum injection time of 50 ms and an automatic gain control (AGC) value of 4 × 10^5^ was used to perform scans. A top-speed acquisition algorithm was used to determine the number of selected precursor ions for fragmentation. Selected precursor ions were isolated in the quadrupole using an isolation width of 1.6 Da. A dynamic exclusion was applied to analysed peptides after 60 s and only peptides with a charge state between 2+ and 7+ were analysed. Precursor ions were fragmented using higher energy collision-induced dissociation with a normalized collision energy of 28%. The resulting MS/MS ions were measured in the linear ion trap. MS/MS scan conditions were typically the following: a targeted AGC value of 2 × 10^4^ and a maximum fill time of 35 ms.

### Differential LC-MS/MS analysis

Raw LC-MS/MS files results were interrogated using Progenesis QI for Proteomics (NonLinear Dynamics, Waters) as described previously [75]. Proteome Discover version 2.1 software (Thermo Scientific) with the SEQUEST HT algorithm was used to identify proteins. The Uniprot CHO database (fasta database downloaded in July 2019 containing 23,959 sequences) was used for protein identification. All Proteome Discover searches had the following criteria applied: (1) precursor mass tolerance set at 20 ppm (2) fragment mass tolerance set at 0.6 Da (3) oxidation of methionine set as a dynamic modification, (4) carbamidomethylation of cysteine set as a static modification, and (5) a maximum of two missed cleavage sites was allowed. A false-discovery rates of < 5% was applied using Percolator. The enzyme specificity was set as trypsin for all samples. Lists of DE proteins were filtered by the following criteria (a) > 1 unique peptide identified in each protein (b) fold change of > ±1.5 (c) ANOVA of < 0.05. An outline of the proteomic experimental workflow is shown in Additional file 2.

### Gene ontology analysis of DE protein lists

The following Gene Ontology (GO) databases were used to analyse all lists of DE proteins; DAVID (https://david.ncifcrf.gov) and STRING (https://string-db.org). Official gene symbols were used to identify protein in GO databases. Databases were used to identify biological functions and molecular processes which were enriched within our lists of DE proteins.

### Statistical analysis

The following statistical analysis was used to determine significance represented on all graphs. A two-tailed student t-test was performed on all phenotypic parameters measured between CDCLs. An F-test was first performed on all data to determine whether equal or unequal variance should be used for the Students t-test. An F statistic of lower value than the critical F value indicated equal variance and an F statistic higher than the critical F value indicated unequal variance. Data with a *p*-value ≤0.05 was considered lowly significant, ≤ 0.005 was considered significant and ≤ 0.001 considered highly significant.

## Supplementary Information

**Additional file 1.** Additional phenotypic profiling of high and low peak VCD CDCLs. (A) IVCD (B) Lactate (C) Glucose (D) Ammonia (E) Glutamine (F) Glutamate (G) Cell size (H) Cell volume (I) Gene copy number (J) Transcript copy number of each individual high and low peak VCD CDCLs over a 17 fed batch shake flask study. Error bars represent the standard deviation of three high peak VCD or three low peak VCD CDCLs, with two biological replicates per CDCL. (* < 0.05, ** < 0.005, *** < 0.001).

**Additional file 2.** Experimental setup/workflow. (A) Summary of CDCLs used for high Vs low peak VCD (experiment 1) and normal Vs extended culture VCD (experiment 2) differential LC-MS/MS proteomic analysis. CDCLs which overlap between experiments are highlighted in yellow (B) Workflow for sample preparation and LC-MS/MS analysis. (PNG)

**Additional file 3.** Differentially expressed proteins identified by LC-MS/MS analysis. Full list of differentially expressed proteins identified between high/low peak VCD and extended/normal VCD CDCLs at day 6 and 10 of culture (xls).

**Additional file 4.** Profiling of high/low peak VCD peak CDCLs throughout the shake flask terminal study. Average (A) VCD, (B) TCD, (C) Cell viability, (D) Titre, (E) Specific productivity, (F) Growth rate (h-1) of each individual high peak VCD and low peak VCD CDCLs. Error bars represent the standard deviation of three high peak VCD or three low peak VCD CDCLs, with two biological replicates per CDCL.

**Additional file 5.** Profiling of extended culture VCD and normal culture VCD CDCLs throughout the shake flask terminal study. Average (A) VCD, (B) TCD, (C) Cell viability, (D) Titre, (E) Specific productivity, (F) Growth rate (h-1) of of each individual normal and extended culture VCD CDCLs. Error bars represent the standard deviation of two extended VCD or two normal VCD CDCLs, with two biological replicates per CDCL.

**Additional file 6.** Additional phenotypic profiling of high and low peak VCD CDCLs. (A) IVCD (B) Lactate (C) Glucose (D) Ammonia (E) Glutamine (F) Glutamate (G) Cell size (H) Cell volume (I) Gene copy number (J) Transcript copy number of each individual extended and normal VCD CDCLs over a 17 fed batch shake flask study. Error bars represent the standard deviation of two extended VCD or two normal VCD CDCLs, with two biological replicates per CDCL. (* < 0.05, ** < 0.005, *** < 0.001).

**Additional file 7.** Principal component analysis (PCA) output from Progenesis Qi for proteomics showing clustering of differentially expressed peptides between; A) high Vs low peak VCD at day 6, B) high Vs low peak VCD at day 10, C) normal Vs extended culture VCD day 6 and D) normal Vs extended culture VCD day 10.

## Data Availability

The datasets generated and/or analysed during the current study are not publicly available due to proprietary information that may be derived publicly from this industrial cell line but are available from the corresponding author on reasonable request.
