# Supplementary material for: Mapping the molecular basis for growth related phenotypes in industrial producer CHO cell lines using differential proteomic analysis
Source: BMC Biotechnol. 2021 Jul 23;21:43. doi: 10.1186/s12896-021-00704-8 (PMC8305936; doi:10.1186/s12896-021-00704-8)
Supplement: Supplementary file 1 — Additional file 1. Additional phenotypic profiling of high and low peak VCD CDCLs. (A) IVCD (B) Lactate (C) Glucose (D) Ammonia (E) Glutamine (F) Glutamate (G) Cell size (H) Cell volume (I) Gene copy number (J) Transcript copy number of each individual high and low peak VCD CDCLs over a 17 fed batch shake flask study. Error bars represent the standard deviation of three high peak VCD or three low peak VCD CDCLs, with two biological replicates per CDCL. (* < 0.05, ** < 0.005, *** < 0.001). [file 12896_2021_704_MOESM1_ESM.docx]

B

D

C

*

**

***

*

A

*

F

E

**

*

*

*

*
