# Supplementary figures and images for: Mapping the molecular basis for growth related phenotypes in industrial producer CHO cell lines using differential proteomic analysis
Source: BMC Biotechnol. 2021 Jul 23;21:43. doi: 10.1186/s12896-021-00704-8 (PMC8305936; doi:10.1186/s12896-021-00704-8)

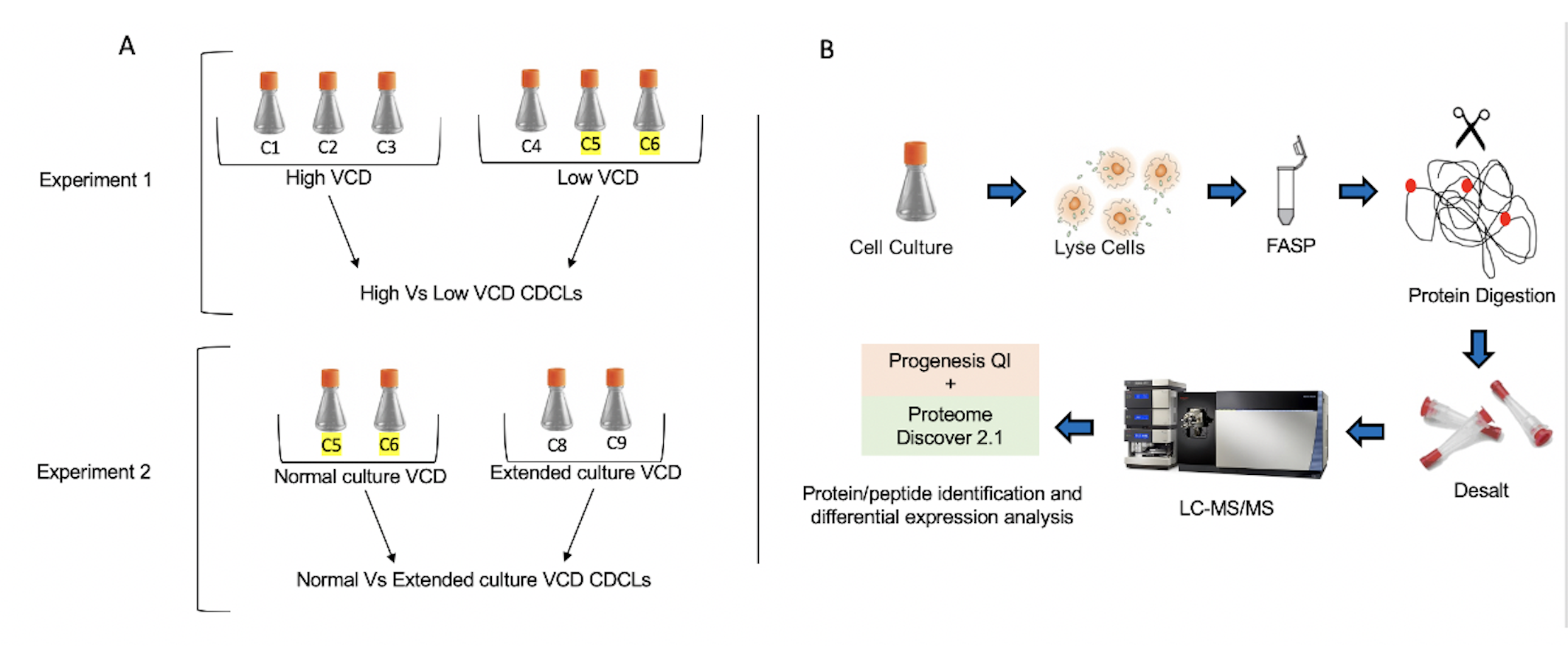

Supplement: Supplementary file 2 — Additional file 2. Experimental setup/workflow. (A) Summary of CDCLs used for high Vs low peak VCD (experiment 1) and normal Vs extended culture VCD (experiment 2) differential LC-MS/MS proteomic analysis. CDCLs which overlap between experiments are highlighted in yellow (B) Workflow for sample preparation and LC-MS/MS analysis. (PNG) [file 12896_2021_704_MOESM2_ESM.png]
