# Supplementary material for: Mapping the molecular basis for growth related phenotypes in industrial producer CHO cell lines using differential proteomic analysis
Source: BMC Biotechnol. 2021 Jul 23;21:43. doi: 10.1186/s12896-021-00704-8 (PMC8305936; doi:10.1186/s12896-021-00704-8)
Supplement: Supplementary file 4 — Additional file 4. Profiling of high/low peak VCD peak CDCLs throughout the shake flask terminal study. Average (A) VCD, (B) TCD, (C) Cell viability, (D) Titre, (E) Specific productivity, (F) Growth rate (h-1) of each individual high peak VCD and low peak VCD CDCLs. Error bars represent the standard deviation of three high peak VCD or three low peak VCD CDCLs, with two biological replicates per CDCL. [file 12896_2021_704_MOESM4_ESM.docx]

C

B

*

***

*

A

*

**

**

D

F

E
