# Supplementary material for: Mapping the molecular basis for growth related phenotypes in industrial producer CHO cell lines using differential proteomic analysis
Source: BMC Biotechnol. 2021 Jul 23;21:43. doi: 10.1186/s12896-021-00704-8 (PMC8305936; doi:10.1186/s12896-021-00704-8)
Supplement: Supplementary file 5 — Additional file 5. Profiling of extended culture VCD and normal culture VCD CDCLs throughout the shake flask terminal study. Average (A) VCD, (B) TCD, (C) Cell viability, (D) Titre, (E) Specific productivity, (F) Growth rate (h-1) of of each individual normal and extended culture VCD CDCLs. Error bars represent the standard deviation of two extended VCD or two normal VCD CDCLs, with two biological replicates per CDCL. [file 12896_2021_704_MOESM5_ESM.docx]

*
