# Supplementary material for: Mapping the molecular basis for growth related phenotypes in industrial producer CHO cell lines using differential proteomic analysis
Source: BMC Biotechnol. 2021 Jul 23;21:43. doi: 10.1186/s12896-021-00704-8 (PMC8305936; doi:10.1186/s12896-021-00704-8)
Supplement: Supplementary file 7 — Additional file 7. Principal component analysis (PCA) output from Progenesis Qi for proteomics showing clustering of differentially expressed peptides between; A) high Vs low peak VCD at day 6, B) high Vs low peak VCD at day 10, C) normal Vs extended culture VCD day 6 and D) normal Vs extended culture VCD day 10. [file 12896_2021_704_MOESM7_ESM.docx]

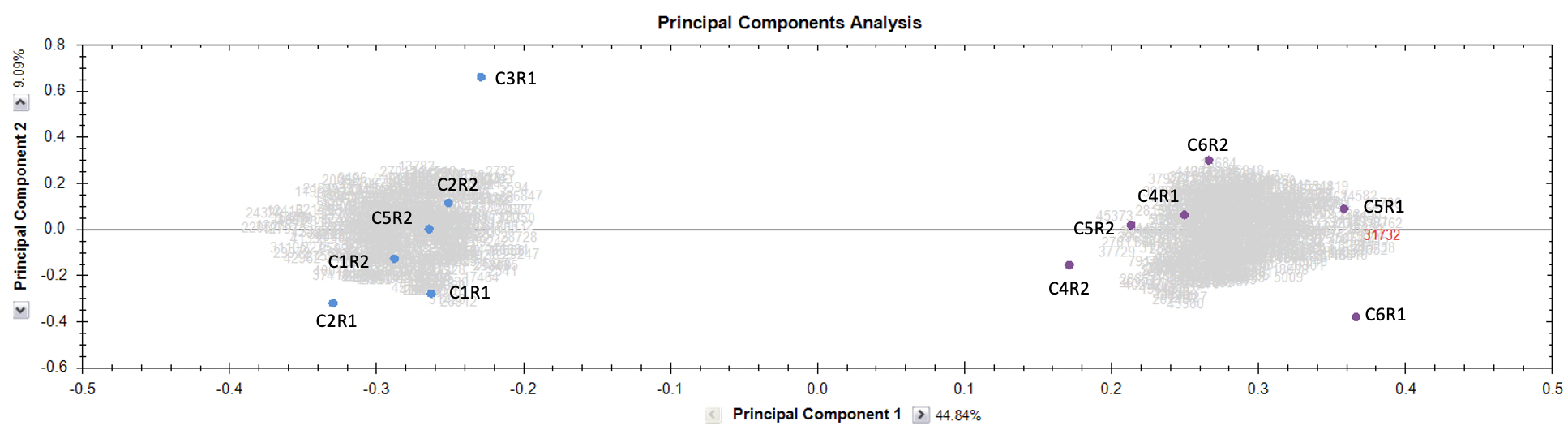


A

B


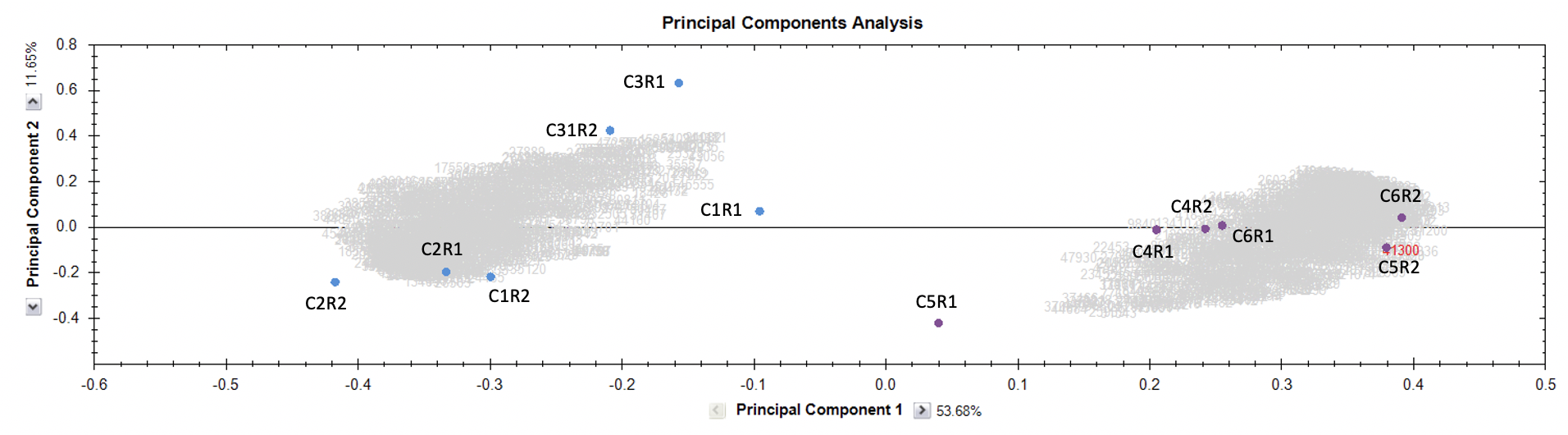


C


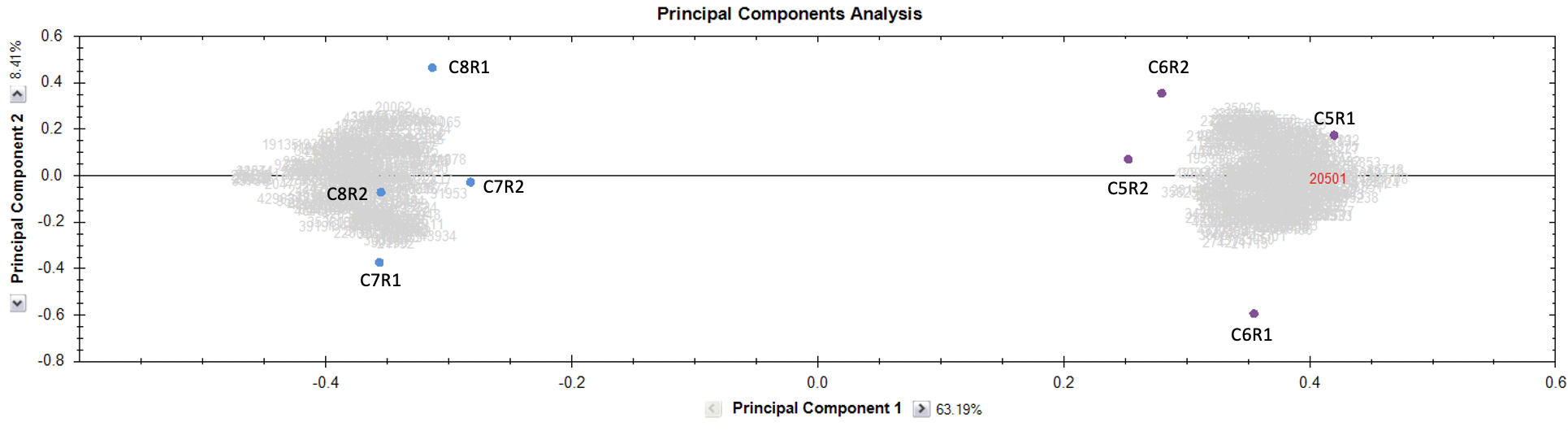


D


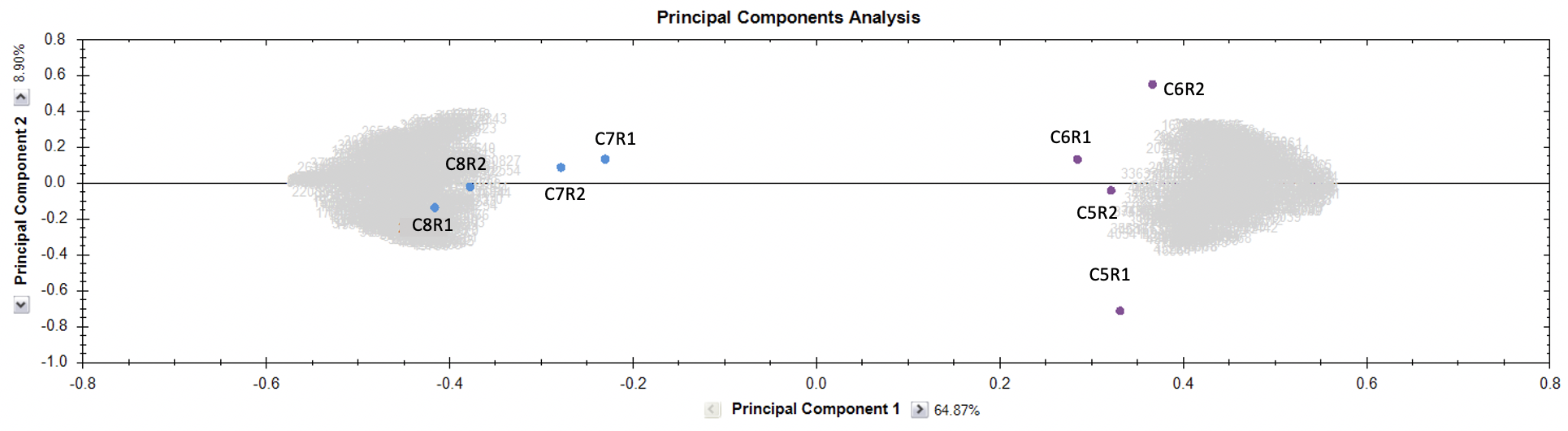


Additional file 5: Principal component analysis (PCA) output from Progenesis Qi for proteomics showing clustering of differentially expressed peptides between; A) high Vs low peak VCD at day 6, B) high Vs low peak VCD at day 10, C) normal Vs extended culture VCD day 6 and D) normal Vs extended culture VCD day 10.
